# Using RNA sequencing to identify putative competing endogenous RNAs (ceRNAs) potentially regulating fat metabolism in bovine liver

**DOI:** 10.1038/s41598-017-06634-w

**Published:** 2017-07-25

**Authors:** Ruobing Liang, Bo Han, Qian Li, Yuwei Yuan, Jianguo Li, Dongxiao Sun

**Affiliations:** 1 Department of Animal Genetics, Breeding and Reproduction, College of Animal Science and Technology, Key Laboratory of Animal Genetics and Breeding of Ministry of Agriculture, National Engineering Laboratory of Animal Breeding, China Agricultural University, Beijing, 100193 China; 2 Department of Animal Production and Environmental Control, College of Animal Science and Technology, Hebei Agricultural University, Baoding, 071001 China

## Abstract

RNA sequencing has been extensively used to study specific gene expression patterns to discover potential key genes related to complex traits of interest in animals. Of note, a new regulatory mechanism builds a large-scale regulatory network among transcriptome, where lncRNAs act as competing endogenous RNAs (ceRNAs) to sponge miRNAs to regulate the expression of miRNA target genes post-transcriptionally. In this study, we sequenced the cDNA and sRNA libraries of nine liver samples from three Holstein cows during dry period, early lactation, and peak of lactation with HiSeq platform. As a result, we identified 665 genes, 57 miRNAs and 33 lncRNAs that displayed differential expression patterns across periods. Subsequently, a total of 41ceRNA pairs (lncRNA-mRNA) sharing 11 miRNAs were constructed including 30 differentially expressed genes. Importantly, 12 among them were presented in our large metabolic networks, and predicted to influence the lipid metabolism through insulin, PI3K-Akt, MAPK, AMPK, mTOR, and PPAR signaling pathways, thus, these genes were considered as the most promising candidates for milk fat formation. To our knowledge, this is first investigation to profile the ceRNA regulatory networks of liver transcriptome that could affect milk fat synthesis in bovine, providing a new view of the regulatory mechanism of RNAs.

## Introduction

Milk and dairy products are an excellent source of rich nutrients for humans, providing protein, fatty acids, minerals, and vitamins^1^. Cow milk production has increased from 0.46 to 0.64 billion tonnes worldwide between 1961 and 2013 (FAOSTAT, Food and Agriculture Organization of the United Nations, 2016, http://faostat3.fao.org/browse/Q/QL/E). Milk production traits are the most important economic traits in the dairy industry. They include milk yield, fat yield, protein yield, fat percentage, and protein percentage^2^. Many researchers have attempted to identify the causal genes that have large effects on milk production traits with the aim of increasing production, including quantitative trait locus (QTL) mapping, candidate gene analysis, and genome-wide association studies (GWAS).

RNA sequencing (RNA-seq) has been used widely to study specific gene expression patterns in different tissues or at different developmental stages, to predict new transcripts^3^, to identify alternative splicing^4^, to detect single nucleotide polymorphisms (SNPs)^5^, and to discover insertion/deletions in transcripts^6^. Several RNA-seq studies of bovine transcriptomes in embryos, liver, mammary glands, and milk have been reported so far^7–15^. Different populations of RNAs have been subjected to RNA-seq, including total RNA^16^, long non-coding RNAs (lncRNAs)^17^, small non-coding RNAs such as microRNAs^18^, ribosomal RNAs^19^, and transfer RNAs^20^. Non-coding RNAs (ncRNAs) that cannot be translated to proteins but can act as functional RNAs have been recognized. The ncRNAs are divided into small RNAs (sRNAs) and lncRNAs depending on their length^21^. MiRNAs were found to be involved in regulating reproductive performances^22^, lactation characters^23, 24^, health^25^, and mammary gland development^26^ in Holstein cows. Regulatory functions of lncRNAs and identification of novel bovine lncRNAs have been reported in horn bud agenesis^27^, pigmented bovine skin^28^, and *longissimus thoracic* ^29^.

A new regulatory mechanism, which involves crosstalk between RNAs (both ncRNAs and coding RNAs) through miRNA response elements (MREs), builds a large-scale regulatory network among transcriptome sequences. LncRNAs may act as competing endogenous RNAs (ceRNAs) to sponge miRNAs, thereby regulating the expression of miRNA target genes post-transcriptionally^30^. The initial experimental evidence for ceRNA crosstalk was the tumor suppressor gene *PTEN* (phosphatase and tensin homolog), which can be regulated by the 3′-UTR of the pseudogene *PTENP1* (phosphatase and tensin homolog pseudogene 1)^31^. Further, *PTEN* ceRNAs have been identified and validated in glioblastoma^32^, melanoma^33^, and prostate cancer^34^. The lncRNA *ADNCR* was shown to inhibit bovine adipocyte differentiation by functioning as a ceRNA for sponging miR-204, thereby increasing the expression of *SIRT1* (sirtuin 1), which is the target of miR-204 and known to repress adipocyte differentiation and adipogenic gene expression^35^. A muscle-specific lncRNA, *lncMD*, functions as a ceRNA to sponge miR-125b, resulting in increasing expression of *IGF2* (insulin like growth factor 2) to promote bovine muscle differentiation^36^.

Liver is a complex digestive gland in ruminant animals including dairy cattle, plays an important role in the metabolism of carbohydrates, fats, proteins, vitamins, hormones, and other substances. Nutrients absorbed from the digestive tract pass through the liver, enter the circulatory system, and finally arrive in the mammary glands of dairy cattle. During the lactation period, liver plays a critical role^37–39^. Dorland *et al*. reported the period of transition from late gestation to early lactation involved considerable metabolic adaptation in dairy cows and the liver, as a key role in adaptation, and supported pregnancy and lactation through coordination and interconversion of nutrients^39^. Smith *et al*. suggested that a part of the adaptation aimed to increase the hepatic pool of cholesterol, which was required for an increased formation of bile acids, and for synthesis and secretion of lipoproteins to provide the mammary gland with cholesterol and triglycerides for milk production^40^. In the present study, we isolated the total RNA and sRNAs from livers of Holstein cows 50 days before (dry period), and 10 days (early lactation) and 60 days (peak of lactation) after parturition for RNA-seq and sRNA-seq, respectively. Interactions among differentially expressed miRNAs, differentially expressed genes, and lncRNAs across the three lactation periods were identified. We built ceRNA regulatory networks via miRNAs to reveal the potential regulatory mechanisms controlling milk fat formation in liver of dairy cattle.

## Results

### Sequencing and mapping of the bovine liver transcriptome

We sequenced the cDNA and sRNA libraries of nine liver samples from three Holstein cows (A, B, and C) during three periods (dry period, −50 days; early lactation, +10 days; peak of lactation, +60 days). We acquired 80,470,996- 99,986,546 paired-end reads 100 bp in length for the mRNAs and lncRNAs, and 10,277,358- 15,107,016 single-end reads 50 bp in length for the miRNAs in the nine samples. After removing reads containing adapters, reads containing poly-N/A, and low quality reads from raw data, we obtained 97.56 gigabases (Gb) and 5.27 Gb high-quality clean data for the mRNAs/lncRNAs and miRNAs, respectively (see Supplementary Table S1). All the clean reads were aligned to the bovine reference genome assembly (UMD3.1.80) for further analysis.

We calculated the correlation coefficient (*r* ^2^) of the sequencing data among the three individual cows in each period based on the FPKM and TPM values, and found that the *r* ^2^ values were 0.860–0.995, 0.973–0.998, and 0.883–0.983 for the mRNAs, miRNAs, and lncRNAs, respectively, indicating that the similarity of the three biological replicates was sufficiently high (see Supplementary Table S2). We also found the average *r* ^2^ between the randomly selected 10 mRNA, 10 miRNA, and nine lncRNA expression levels from the qRT-PCR and the corresponding sequencing data were 0.72, 0.66, and 0.71 respectively, confirming the high reproducibility of the sequencing data in this study (see Supplementary Fig. S1).

### Identification and function analysis of differentially expressed genes, miRNAs, and lncRNAs

We identified a total of 20,831 mRNAs corresponding to 16,857 known and 1,665 putative novel genes, 1,046 miRNAs (446 known and 600 novel with miRDeep2 scores ≥1.0), and 1,704 lncRNAs. Of these, totally 665 genes, 57 miRNAs, and 33 lncRNAs were found to be differentially expressed between at least two of the periods using Cuffdiff (v2.1.1) (Table 1; see Supplementary Table S3).

**Table 1:** Differentially expressed RNAs during dry period, early lactation, and peak of lactation.

RNA name	Known/novel	Early lactation vs. Dry period		Peak of lactation vs. Dry period		Peak of lactation vs. Early lactation		Total
		Up	Down	Up	Down	Up	Down	
mRNA	Known	236	173	189	106	102	62	598
	Potential	19	34	19	11	16	5	67
miRNA	Known	8	0	5	1	1	2	12
	potential	9	4	22	1	29	3	45
lncRNA	Known	0	1	0	0	0	0	1
	potential	6	13	5	9	5	1	32

Up means that the expression of mRNAs, miRNAs, or lncRNAs were up-regulated, and down means the expression of mRNAs, miRNAs, or lncRNAs were down-regulated.

By scanning for conserved miRNA target sites with miRanda and RNAhybrid, we predicted 10,749 target genes for the differentially expressed miRNAs in common. In addition, we searched coding genes within the 100-kb upstream and downstream regions of each differentially expressed lncRNA and found 76 cis-acting genes. We also calculated the expression correlation coefficients between the differentially expressed lncRNAs and all identified mRNAs, and detected 31 lncRNAs associated with 852 potential trans-regulated genes (see Supplementary Table S4).

Further, we performed functional enrichment analysis on these differentially expressed genes (DEGs) and target genes of differentially expressed miRNAs and lncRNAs. As a result, GO categories and pathways for DEGs such as fatty acid, amino acid, carbohydrate and energy metabolism, immune response, PPAR, MAPK, AMPK, and PI3K-Akt signaling pathways were clustered in functional enrichment analysis (see Supplementary Tables S5 and 6).

### Construction of ceRNA regulatory networks for milk composition

**Figure 1:**
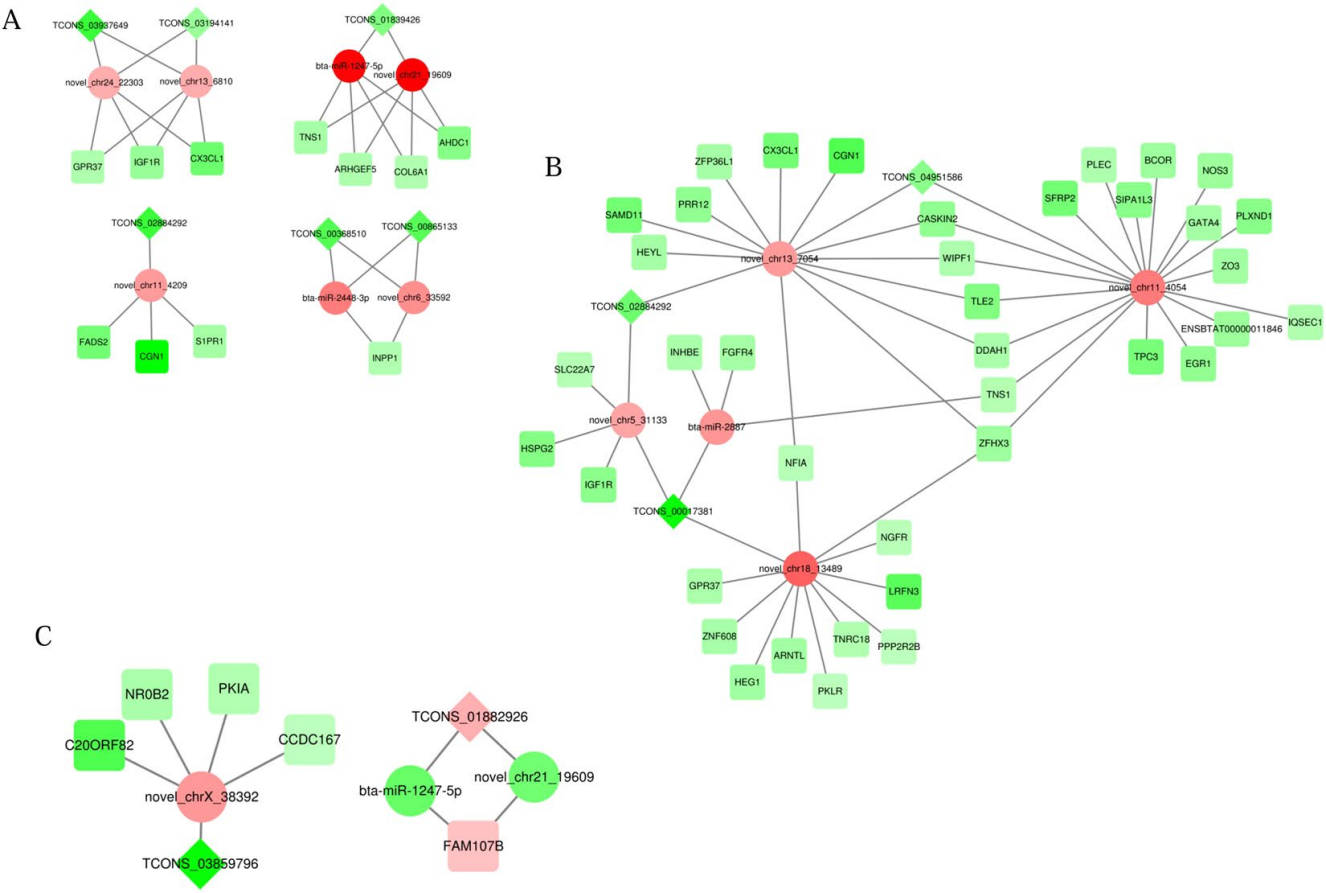
ceRNA regulatory networks among dry period, early lactation, and peak of lactation. (**A**) Early lactation vs. dry period; (**B**) peak of lactation vs. dry period; (**C**) peak of lactation vs. early lactation; red circles, squares and diamonds represent up-regulated miRNAs, mRNAs and lncRNAs, respectively; and the green circles, squares and diamonds represent down-regulated miRNAs, mRNAs and lncRNAs, respectively.

**Figure 2:**
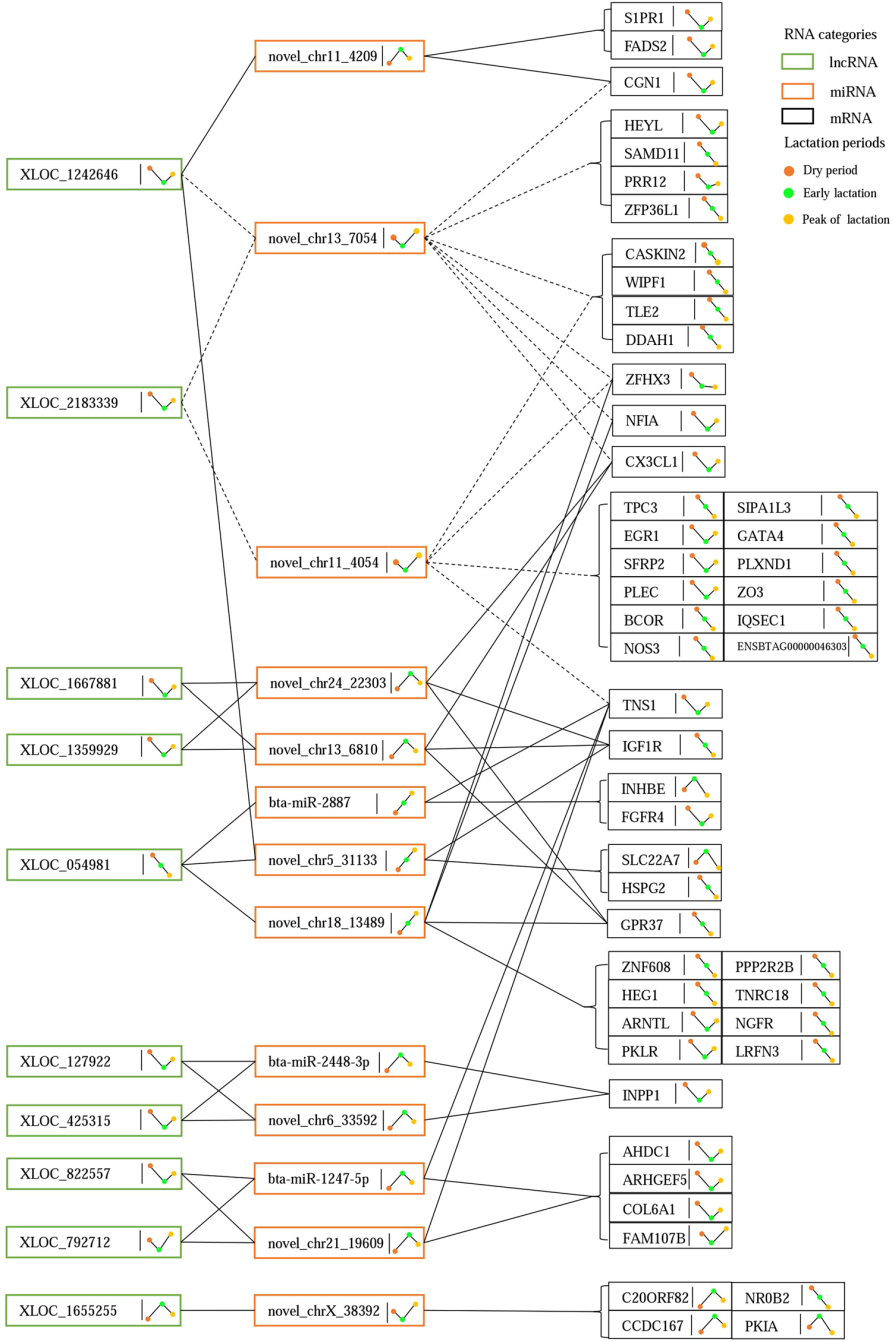
Definite expression of RNAs in ceRNA networks profiling their dynamic expression trends. The definite expression of lncRNAs, miRNAs, and mRNAs are shown based on FPKM, TPM, and FPKM values, respectively; green, orange, and black rectangles represent lncRNAs, miRNAs, and mRNAs, respectively; red, green, and yellow dots represent dry period, early lactation, and peak of lactation, respectively; the lines linked by these color dots show the dynamic expression trends of RNAs during periods; the full lines mean that interactions between RNAs follow the ceRNA regulatory mechanism; and the dash lines mean that the interactions between RNAs do not obey the ceRNA regulatory mechanism.

Among the predicted targets for 57 differentially expressed miRNAs, 251 DEGs were included targeted by 47 miRNAs. Subsequently, we scanned potential MREs in the 33 differentially expressed lncRNAs specifically targeted by the differentially expressed miRNA using miRanda v3.3a, and found 47 miRNA–lncRNA interaction pairs. Further, we constructed ceRNA regulatory networks that indicated the cross-regulations among lncRNA–miRNA–mRNA via shared miRNAs by calculating the likelihood of each potential ceRNA pair (lncRNA-mRNA) based on a hypergeometric test (*P* < 0.05). Consequently, we obtained 80 ceRNA pairs, including 16 between the dry period and early lactation, 59 between the dry period and peak of lactation, and five between the early and peak of lactation periods (Fig. 1; see Supplementary Table S7), in which lncRNAs competitively bound miRNAs thereby up-regulated the targeting mRNA expression, reversely, lncRNAs decreased the mRNA expression by releasing MREs. Further, we profiled the dynamic expression trends of all DEGs, miRNAs and lncRNAs in the identified ceRNA networks among three periods based on their FPKM and TPM values, and finally determined 41 ceRNA pairs that shared 11 miRNAs (Fig. 2). Two miRNAs (novel_chr13_7054 and novel_chr11_4054) and their targeting lncRNAs and genes which showed almost same expression trends were ruled out (Fig. 2). In total, 30 DEGs, 11 miRNAs and 9 lncRNAs were included in the ceRNA networks.

### Construction of lipid metabolic network regulated by ceRNAs

**Figure 3:**
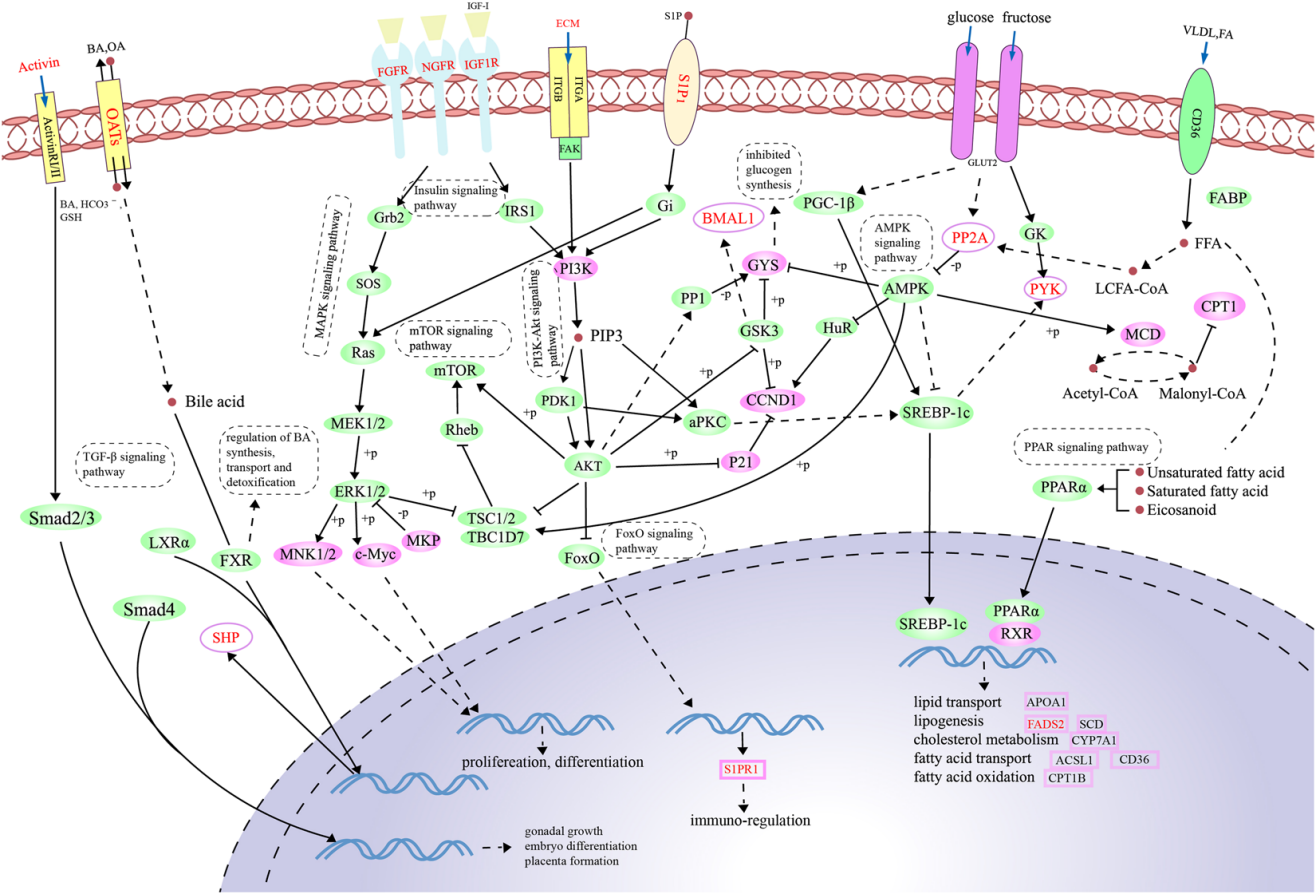
Metabolic networks involving in insulin, PI3K-Akt, AMPK, MAPK, mTOR, FoxO, and PPAR signaling pathways. Green ovals represent proteins involved in pathways; pink ovals (rectangles) represent the differentially expressed proteins (genes) identified in this study; the 12 genes in ceRNA networks are marked in red font, of these, BMAL1, ECM, IGF1R, NGFR, Activin, FGFR, PYK, SHP, S1P_1_, OATs, and PP2A protein in the networks respectively encoded by *ARNTL*, *COL6A1*, *IGF1R*, *NGFR*, *INHBE*, *FGFR4*, *PKLR*, *NR0B2*, *S1PR1*, *SLC22A7* and *PPP2R2B* genes, and the remaining *FADS2* gene is in the pink rectangles.

**Figure 4:**
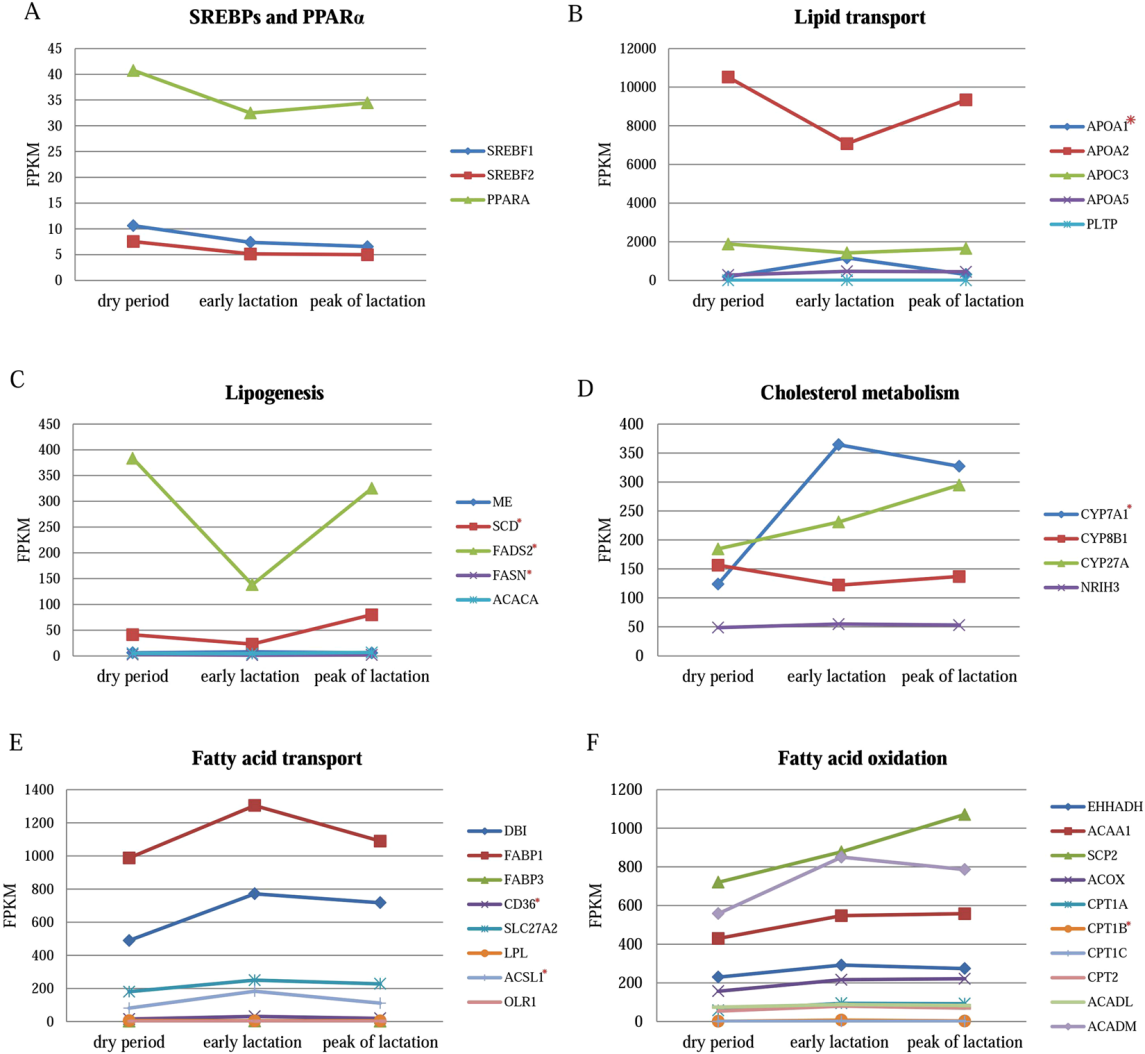
Expression profiles of the lipid metabolic genes during dry period, early lactation, and peak of lactation. The expression levels of lipid metabolic genes are shown based on FPKM values; the genes are divided into SREBPs and PPARα (**A**), lipid transport (**B**), lipogenesis (**C**), cholesterol metabolism (**D**), fatty acid transport (**E**), and fatty acid oxidation (**F**) categories; and the differentially expressed genes are labeled with an asterisk.

We built a metabolic network comprising 12 out of 30 DEGs in ceRNA network that were involved in insulin, PI3K-Akt, AMPK, MAPK, mTOR, FoxO, and PPAR signaling pathways (Fig. 3; see Supplementary Table S8). As well-known, sterol regulatory element-binding proteins (SREBPs) and PPARα occupy the critical position in the lipid metabolism^41, 42^. Membranous receptors can influence SREBP-1c or PPARα through insulin, PI3K-Akt, AMPK, and PPAR pathways thereby modulating expression of lipid metabolic genes. As shown in Fig. 3, four membranous receptor genes (*IGF1R*, *FGFR4*, *NGFR*, and *S1PR1*) and *COL6A1* in our ceRNA networks were down-regulated by six lncRNAs (Fig. 2), and induced the decreasing expression of *PIK3R1*, a critical node in PI3K-Akt pathway, so that SREBP-1c expression was correspondingly decreased through metabolic cascades. In addition, *PPP2R2B*, which was down-regulated by lnc-*XLOC_054981*, reduced SREBP-1c expression as well by increasing AMPK expression. Besides, the elevating AMPK up regulated the expression of CPT1 leading to the fatty acid oxidation through the differentially expressed MCD, and the decreasing PI3K modulated the expression of *S1PR1* to affect the immune-regulation through PI3K-Akt and FoxO signaling pathways. Moreover, IGF1R, FGFR4, NGFR, and SIP_1_ protein reduced the expression of MNK1/2 and c-Myc via insulin, PI3K-Akt, and MAPK signaling pathways to modulate the proliferation and differentiation. The differentially expressed activin affected gonadal growth, embryo differentiation, and placenta formation through TGF-β signaling pathway. Bile acids entered into the cytoplasm through the receptor OATs that expressed differentially, and interacted with FXR to regulate the bile acid synthesis, transport, and detoxification, meanwhile, the nuclear receptor FXR also led to the differentially expression of SHP. Fatty acid and very low-density lipoprotein were transported into cytoplasm through CD36, and the unsaturated or saturated fatty acids and eicosanoid activated the expression of PPARα, which combined with RXR to modulate the expression of the lipid metabolic genes. As shown in Fig. 4, the downstream genes were up-/down-regulated by SREBP-1c and PPARα, among these, *APOA1* (apolipoprotein A1), *ACSL1* (acyl-CoA synthetase long-chain family member 1), *CD36* (CD36 molecule), *CYP7A1* (cytochrome P450, family 7, subfamily A, polypeptide 1), *CPT1B* (carnitine palmitoyltransferase 1B), *SCD* (stearoyl-CoA desaturase) and *FADS2* (fatty acid desaturase 2) were evidenced to be differentially expressed, and eventually influenced the lipid and fatty acid transport, cholesterol metabolism, fatty acid oxidation, and lipogenesis, which belong to lipid metabolism in liver (see Supplementary Table S9).

Based on biological functions, 12 among the 30 genes in the ceRNA networks were considered as keys participating in important lipid metabolic processes in liver, that were *ARNTL* (aryl hydrocarbon receptor nuclear translocator-like), *COL6A1* (collagen, type VI, alpha 1), *IGF1R* (insulin like growth factor 1 receptor), *NGFR* (nerve growth factor receptor), *INHBE* (inhibin, beta E), *FGFR4* (fibroblast growth factor receptor 4), *PKLR* (pyruvate kinase, liver and RBC), *FADS2* (fatty acid desaturase 2), *NR0B2* (nuclear receptor subfamily 0 group B member2), *S1PR1*(sphingosine-1-phosphate receptor 1), *SLC22A7* (solute carrier family 22 member 7) and *PPP2R2B* (protein phosphatase 2 regulatory subunit Bbeta).

## Discussion

We investigated the complexity of the liver transcriptome in Holstein cows at three levels, mRNA, miRNA, and lncRNA, using high-throughput RNA sequencing. We detected 665 genes, 57 miRNAs, and 33 lncRNAs that had different expression patterns among the dry period (−50 day), early lactation (+10 day), and peak of lactation (+60 days). Based on these results, we constructed 41 ceRNA pairs containing 30 DEGs, among which 12 genes were considered as the most important ones participating in lipid metabolism in liver of dairy cattle.

In this study, besides 665 DEGs, we identified a total of 10,749 target genes for 57 differentially expressed miRNAs, and 76 cis- and 852 trans-targets for 33 differentially expressed lncRNAs. By performing functional enrichment analysis for these genes, it was found that GO categories and pathways for DEGs such as fatty acid, amino acid, carbohydrate and energy metabolism, immune response, PPAR, MAPK, AMPK, and PI3K-Akt signaling pathways were enriched among three periods. Furthermore, genes including DEGs, and target genes of differentially expressed miRNAs between the dry period and early and/or peak of lactation were enriched mainly in metabolism-related functions, especially in processes involving carbohydrates, lipids and protein processes, suggesting that the genes regulating substance synthesis remained active to support the synthesis of a large number of milk compositions in both early and peak of lactation. Here, we found several pathways related to carbohydrate metabolism between dry period and early and/or peak of lactation including pyruvate metabolic process, glucocorticoid metabolic process, and regulation of glycogen catabolic process, and a study showed that gluconeogenesis in liver is vital to meet glucose requirements for dairy cows in perinatal period^43^. Additionally, fatty acids can be re-esterified to triglycerides in the liver of dairy cows^44^. The contents of triacylglycerol^45^ and blood nonesterified fatty acids (NEFA)^46^ were increased greatly during the perinatal period, and Pullen *et al*. reported that the free fatty acid absorption in liver was directly in proportion to NEFA^47^. Kristensen *et al*. suggested that milk fat proportion depended on the precursors freed by liver, including propionic acid, branched chain volatile fatty acids and mid/long chain fatty acids^48^. Our study also found that some genes were enriched into lipid metabolism in early and peak of lactation compared with dry period, such as bile secretion, biosynthesis of unsaturated fatty acids, fatty acid synthase activity, lipid homeostasis, carboxylic acid metabolic process, fatty acid metabolism, PPAR, and AMPK signaling pathways.

The recognition of ceRNAs has opened a new way of analyzing the role of RNAs in gene regulation. Here, we first constructed ceRNA networks across three periods in liver of dairy cows based on the differentially expressed mRNAs, miRNAs, and lncRNAs. In ceRNA networks, miRNAs targets RNA transcripts through MREs, which are approximately 22 nt in length and can be partially complementarity for target RNAs. If an RNA transcript has numerous MREs, miRNAs can function in a combinatorial manner^30^. As our ceRNA results showed, some lncRNA-mRNAs interacted with each other via several different miRNAs, and the expression trends of these pairs were the same, while opposite to the expression tendency of miRNAs. On the other hand, a single miRNA can also simultaneously target multiple lncRNAs or mRNAs. Such phenomenon indicated that a miRNA occupied the central position by supplying multiple intermediate bridges for lncRNAs and mRNAs. Li *et al*. first predicted genome-wide miRNA-mRNA and miRNA-lncRNA interactions in chicken, pigs and cows using the sequencing data from public databases, and found many SNPs located in miRNAs that may affect the interactions between miRNAs and mRNAs^49^. Li *et al*. and Sun *et al*. reported that lncRNAs act as ceRNAs to module mRNA expression by sponging miRNA in bovine adipocyte differentiation^35^ and muscle differentiation^36^, respectively. In liver cancer, Wang *et al*. demonstrated that lnc-*HULC* up-regulated cAMP responsive element binding (*CREB*) expression by interaction with miR-372^50^, and Li *et al*. found lnc-*HULC* functioned as a ceRNA to up-regulate zinc finger E-box binding homeobox 1(*ZEB1*)^51^. Our findings evidenced that 9 lncRNAs acted as ceRNAs to affect the expression of lipid metabolic genes by sponging miRNAs in bovine liver. Interestingly, we found that some lncRNAs might play predominant roles on affecting gene expression in the complicated ceRNA regulatory networks. As an example, *IGF1R* was a common target of three miRNAs which could combine with four lncRNAs as well; however, the lnc-*XLOC_054981* mainly influenced *IGF1R* expression by sponging the miRNA (novel_chr5_31133).

Regarding the constructed ceRNA networks, we preferentially desired to explore their roles in milk composition formation. Based on biological functions of the 30 genes in ceRNAs, we constructed a large complicated metabolic network, and found that 12 out of 30 genes played critical roles in metabolism through several pathways, in which SREBP-1cand PPARα were the two keys in liver for lipid metabolism. Previous studies reported that SREBPs integrated signals from multiple pathways to control fatty acids, triglycerides, and cholesterol synthesis^52, 53^. PPARα, enriched in liver, was involved in peroxisomal and mitochondrial β-oxidation, fatty acid transport and catabolism, hepatic glucose production, lipogenesis and ketone body synthesis^42^. Our results also showed that the lipid metabolic genes involved in lipid transport, fatty acid oxidation and transport, and cholesterol metabolism were up-regulated since lactating, while the genes participating in lipogenesis were down-regulated, indicating that more fatty acids were transported to mammary gland for milk fat production since lactating, but the lipid synthesis in liver dropped off in order to meet homeostasis. Therefore, the 12 genes were inferred as the most promising candidate genes affecting milk fat formation, which were further described as follows.

*IGF1R* combines with insulin-like growth factor (*IGF1*). It was shown to be associated with milk production traits in dairy cattle^54–56^. In our previous GWAS, *IGF1R* was also included as one of promising genes for fatty acid traits in Chinese Holstein^57^. The protein encoded by *FGFR4* is a member of the fibroblast growth factor receptor (FGFR) family. Previous studies found that integrated *FGF15*/*FGFR4* and *SHP* signaling pathways regulated bile acid synthesis in the gut-liver axis^58^, and *FGFR4* exerted a negative control on cholesterol metabolism and bile acid synthesis in liver^59^. *NR0B2*, also known as small heterodimer partner (*SHP*), is a member of the nuclear hormone receptor family and is involved in lipid homeostasis by regulating the expression levels of several genes in the lipid pathway^60, 61^. SNPs in *NR0B2* was found to be associated with carcass- and fat-related traits in beef cattle^62^. As noted above, the integrated *FGF15*/*FGFR4* and *SHP* signaling pathways show there is a relationship between *FGFR4* and *SHP* (*NR0B2*). The protein encoded by *PKLR* is a pyruvate kinase that participates in the glycolysis and gluconeogenesis metabolic pathways (http://www.wikipathways.org/index.php/Pathway:WP534). A report showed that the high long-chain n-3 fatty acid in rat milk associated with low hepatic *PKLR* expression was relevant to hepatic metabolic regulation in a milk-fed neonate^63^. *FADS2*, belonging to the fatty acid desaturase (FADS) family, was involved in fatty acid synthesis and desaturation^64–68^. *S1PR1*, also known as endothelial differentiation gene 1 (*EDG1*), encodes a protein that is structurally similar to G protein-coupled receptors and is highly expressed in endothelial cells^69^. SNPs in the untranslated regions of *EDG1* were related to marbling in Japanese Black beef cattle^70^. *ARNTL* (*BMAL1*) has been identified as a candidate gene for susceptibility to hypertension, diabetes, and obesity, and mutations in *BMAL1* have been linked to infertility, gluconeogenesis and lipogenesis problems, and altered sleep patterns^71, 72^. Another study shows that the CLOCK/BMAL1 complex up regulates human LDLR promoter activity, suggesting the *ARNTL* gene also plays a role in cholesterol homeostasis^73^. Collagen VI is a major structural component of microfibrils, which is a heterotrimer of COL6A1, COL6A2, and COL6A3 protein. It was reported that the collagens, a superfamily of proteins, played a role in maintaining the integrity of various tissues and collagen IV is an important component of the extracellular matrix in the mammary glands^74^. *NGFR* plays a role in the regulation of the translocation of GLUT4 to the cell surface in adipocytes in response to insulin, and thereby contributes to the regulation of insulin-dependent glucose uptake^75^. *INHBE* may be up-regulated under conditions of endoplasmic reticulum stress^76^, and this protein may inhibit cellular proliferation^77^. *SLC22A7* belongs to the solute carrier (SLC) family and is a liver-specific expressed gene^78, 79^. The protein encoded by *PPP2R2B* belongs to the phosphatase 2 regulatory subunit B family. Moreover, we identified that the 12 genes were markedly enriched into PPAR, MAPK, mTOR, insulin, AMPK, PI3K-Akt, and FoxO signaling pathways. Evidently, these 12 genes regulated by ceRNAs mechanism might be the crucial regulatory factors for milk fat formation in liver of dairy cattle.

## Conclusions

In this study, we built a total of 41 ceRNA pairs with integrated analysis of the differentially expressed mRNAs, miRNAs, and lncRNAs across the three periods, and 12 functional genes in these ceRNA networks enriched in lipid metabolism were selected as the most promising candidates for milk fat formation. To our knowledge, this is first investigation to uncover the ceRNA regulatory networks of liver transcriptome that could affect milk fat synthesis in bovine.

## Material and Methods

### Ethics statement

All experiments, including all protocols for collection of the liver tissues of experimental individuals and phenotypic observations, were performed in accordance with relevant guidelines and regulations of the Institutional Animal Care and Use Committee (IACUC) at China Agricultural University (Permit Number: DK996) who have reviewed and approved the experiments. Samples were collected specifically for this study following standard procedures with the full agreement of the Hongda Dairy Farm in Mancheng of Hebei Province who owned the animals.

### Samples collection

We selected three Chinese Holstein cows in their 2nd lactation from 1,300 Holstein cows maintained in the Hongda Dairy Farm in Mancheng, Hebei Province, China. To keep minimum variation between cows, the selection of similar milk production was based on their 305-day milk yield, milk protein percentage and milk fat percentage, which was calculated based on 10 test-day records in one lactation period using a multiple trait random regression test-day model by the Dairy Data Center of China (http://www.holstein.org.cn/). The 305-day milk yield, and milk fat and protein percentages in the milk of the three cows were 10,254–11,954 kg, 3.1–3.7%, and 2.7–2.9%, respectively.

We collected liver tissues using puncture biopsy in three periods: dry period (−50 days), early lactation (+10 days), and peak of lactation (+60 days) from each of the three cows. A 1.5–2.0 cm incision using a sterile scalpel blade was made between the 11th and 12th rib on the right side of each cow after injecting with 30 mL of 2% procaine on the sampling position as local anesthesia (China Agricultural University Veterinary Teaching Hospital). Following the skin incision, pressure was applied to the wound with sterile gauze until visual signs of the bleeding were gone. The liver biopsy was performed using a 4-mm Tru-Cut biopsy needle (Wuhan Anscitech Farming Technology Co. Ltd. Wuhan, China). The skin incision was closed using four or five Michel clips and erythromycin ointment was applied to the incision site. The liver tissue samples (about 0.5–1.0 g) per cow in every period were collected carefully for RNA isolation, placed into clean RNAase-free Eppendorf tubes, and stored in liquid nitrogen.

### RNA isolation and quality assessment

We used TRIzol reagent (Life Technologies, CA, USA) to extract total RNA from the nine liver samples. RNase-free DNase (TIANGEN, Beijing, China) was used to remove DNA contamination from the extracted RNA. RNA degradation and contamination was monitored on 1% agarose gels and RNA purity was checked using a NanoPhotometer^®^ spectrophotometer (IMPLEN, CA, USA). A Qubit^®^ RNA Assay Kit in Qubit^®^ 2.0 Fluorometer (Life Technologies) was used to measure the RNA concentration and a RNA Nano 6000 Assay Kit on a Bioanalyzer 2100 system (Agilent Technologies, CA, USA) was used to assess the RNA integrity.

### RNA sequencing

#### mRNA and lncRNA library construction

A total of 3 μg RNA per sample was used for RNA sample preparation. The RNA integrity numbers of the nine samples from three cows in the three periods ranged from 7.3 to 7.8. Firstly, ribosomal RNA (rRNA) was removed using an EpicentreRibo-Zero™ rRNA Removal Kit (Epicentre, WI, USA) and the rRNA-free residue was obtained by ethanol precipitation. Subsequently, the sequencing libraries were generated using the rRNA-free RNA with a NEBNext^®^ Ultra™ Directional RNA Library Prep Kit for Illumina^®^ (New England Biolabs (NEB), MA, USA). The library fragments were purified with an AMPure XP system (Beckman Coulter, Beverly, USA) and cDNA fragments, preferentially 150–200 bp in length, were selected. Then, 3 μL USER Enzyme (NEB) was used with the selected adaptor-ligated cDNA at 37 °C for 15 min followed by 5 min at 95 °C before PCR. The PCRs were performed with Phusion High-Fidelity DNA polymerase, Universal PCR primers, and Index (X) Primer. The PCR products were purified (AMPure XP system) and library quality was assessed on an Agilent Bioanalyzer 2100 system.

#### miRNA library construction

From the same total RNA samples, a total of 10 μg RNA per sample was used for sRNA sample preparation. Firstly, sRNA was isolated from the total RNA with PEG8000 (Berry Genomics, Beijing, China). Then, the sequencing libraries were generated using a TruSeq Small RNA Sample Prep kit (Illumina, San Diego, CA, USA). A 3′ linker was ligated to the sequences and fractions of sRNAs 36–44 nt in length were extracted from the samples on a 15% denaturing polyacrylamide gel. The short RNAs were converted to DNA by reverse transcription-polymerase chain reaction (RT-PCR) after ligating a 5′ adaptor. The library fragments were purified on 3.5% agarose gels and cDNA fragments, preferentially 140–160 bp in length, were selected. The quality of the library products was assessed on a StepOnePlus PCR system (Applied Biosystems Inc., CA, USA).

#### Clustering and sequencing

For the lncRNAs and mRNAs, a TruSeq PE Cluster Kit v3-cBot-HS (Illumina) was used to cluster the index-coded samples. After cluster generation, the libraries were sequenced on a HiSeq. 2000 platform (Illumina) and 100-bp paired-end reads were obtained.

For the miRNAs, the index-coded samples were clustered using a HiSeq Rapid SBS Kit v2 (Illumina). Then, the libraries were sequenced on a HiSeq. 2500 platform (Illumina) and 50-bp single-end reads were obtained.

### lncRNA and mRNA data analysis

#### Quality control and transcriptome assembly

Firstly, the raw reads in FASTQ format were processed with in-house Perl scripts. In this step, clean reads were obtained by removing reads containing adapters and poly-N, and low quality reads from the raw reads. The Q20 (proportion of bases with a Phred base quality score >20), Q30 (proportion of bases with a Phred base quality score >30), and GC content of the clean data were calculated. All subsequent analyses were based on the high-quality clean data. The bovine reference genome (UMD3.1.80) and gene model annotation files were downloaded from the Ensembl genome website (ftp://ftp.ensembl.org/pub/release-80). An index of the reference genome was built using Bowtie v2.0.6^80^ and paired-end clean reads were aligned to the reference genome using TopHat v2.0.9^81^. The mapped reads of each sample were assembled by both Scripture (beta2)^82^ and Cufflinks (v2.1.1)^6^ using a reference-based approach.

#### Coding potential analysis and target gene prediction for lncRNAs

The following four steps were used to select candidate lncRNAs for classification: (1) Transcript length ≥200 and exon number ≥2; (2) Minimal reads coverage ≥3 in at least one sample; (3) Filter known non-lncRNA annotations; (4) Classify the selected candidate lncRNAs, using four tools to predict their coding potential: CNCI (Coding-Non-Coding-Index) (v2) with default parameters^83^, CPC (Coding Potential Calculator) (0.9-r2) with the e-value set as ‘1e-10’^84^, Pfam Scan (v1.3) with default parameters of -E 0.001 (release 27; both Pfam A and Pfam B were used)^85^, and PhyloCSF (phylogenetic codon substitution frequency) (v20121028) with default parameters^86^. Transcripts predicted to have coding potential by all of the four prediction tools were filtered out, and transcripts with no coding potential were selected as the candidate set of lncRNAs.

To identify cis-acting lncRNAs (lncRNAs that act on neighboring genes), we searched for coding genes within the 100-kb upstream and downstream regions of the selected lncRNAs and then analyzed their functions. Additionally, we calculated the Pearson correlation between the differentially expressed lncRNAs and identified coding genes, and the potential trans-acting genes were selected based on the absolute value of correlation was higher than 0.95.

### miRNA data analysis

#### Quality control and miRNA identification

We used the FASTX software (Fastx_toolkit-0.0.13.2) to pre-process the raw data as follows: remove low quality reads, remove 5′ joint pollution reads, remove 3′ splice reads and no insert fragment reads, remove reads <18-nt long, and remove reads that contain poly-A. The resultant clean reads (sRNAs) were compared against the known ncRNAs (i.e., rRNA, tRNA, snRNA, and snoRNA) in the Rfam database (v10.1) using BLAST (v2.2.26)^87^. The sRNAs that matched any of the sequences in Rfam database were removed. Then, the proportion of the four bases (A/T/G/C) in each position on the clean reads was calculated.

#### Mapping to the reference genome and target gene prediction

The remaining sRNAs that aligned to the bovine reference genome (UMD3.1.80) using TopHat v2.0.9^81^ were searched against the known mature bovine miRNA sequences downloaded from miRBase (http://www.mirbase.org/) to identify known miRNAs. Novel miRNAs were predicted using mirDeep2 (v2.0.0.5) software^88^. To identify candidate target genes, we scanned for conserved miRNA target sites on the mRNAs identified in this study using miRanda (v3.3a)^89^ and RNAhybrid^90^.

### Identification of differentially expressed lncRNAs, mRNAs, and miRNAs

We used Cuffdiff (v2.1.1) to calculate fragments per kilo-base of exon per million mapped fragments (FPKM) values for the lncRNAs and coding mRNAs (genes), and TPM (transcripts per million) scores for the miRNAs in each library^6, 91^. Gene FPKMs were computed by summing the FPKMs of transcripts in each lncRNA or mRNA group. The differential expression of the miRNAs was analyzed using the EdgeR software package^92^. We used the three cows as biological replicates and found differentially expressed lncRNAs, mRNAs, and miRNAs by comparing one period with another. MiRNAs with *P*-values < 0.05, absolute fold-change values >2.0, and miRDeep2 score ≥1, lncRNAs and mRNAs with corrected *P*-values < 0.05 in any of the pairwise comparisons were considered as significantly differentially expressed.

### Quantitative real-time PCR (qRT-PCR)

To validate the repeatability and reproducibility of the RNA sequencing data, qRT-PCR was carried out on 10 DEGs, nine lncRNAs, and 10 miRNAs that were selected randomly using the total RNA that was used for sequencing. Primer3 (http://fokker.wi.mit.edu/primer3/input.htm) and Oligo 6.0 were both used to design specific primers (see Supplementary Table S9). The expression levels of the selected mRNAs and lncRNAs were normalized against the housekeeping gene, *GAPDH*, *UXT* and *RPS9* and the expression levels of the miRNAs were normalized against U6 snoRNA. The qRT-PCRs were carried out in triplicate with the DyNAmo SYBR Green PCR kit (Applied Biosystems Inc.) on a LightCycler480 (Roche Applied Science, Penzberg, Germany) using the following program: 95 °C for 10 min; 45 cycles of 95 °C for 10 s, 60 °C for 10 s, and 72 °C for 10 s; 72 °C for 6 min.

### Function enrichment analysis

We used the KOBAS software^93^ and SetRank^94^ for the Gene Ontology (GO) enrichment and Kyoto Encyclopedia of Genes and Genomes (KEGG) pathway analyses of the differentially expressed genes (DEGs) and predicted target genes of the differentially expressed lncRNAs and miRNAs. Both GO terms and KEGG pathways with corrected *P*-values < 0.05 were considered to be significantly enriched.

### Construction of ceRNA networks

Differentially expressed lncRNAs were scanned to find conserved MREs using miRanda v3.3a, as was done to predict the miRNA–mRNA target site. Based on the predicted miRNA–mRNA and miRNA–lncRNA regulatory pairs that were differentially expressed among the three periods, ceRNA networks were predicted in which lncRNAs and mRNAs interacted via shared miRNAs. We measured the likelihood of the ceRNAs using StarBase v2.0^95–97^. Using the hypergeometric test^32^, we calculated the *P*-value for each potential ceRNA pair (lncRNA-mRNA) considering the number of shared miRNAs targeting individual components of the ceRNA pair with the following formula^97^:

$${\rm{P}}=\sum _{{\rm{i}}={\rm{c}}}^{{\rm{\min }}({\rm{K}},{\rm{n}})}\frac{(\begin{array}{c}{\rm{K}}\\ {\rm{i}}\end{array})(\begin{array}{c}{\rm{N}}-{\rm{K}}\\ {\rm{n}}-{\rm{i}}\end{array})}{(\begin{array}{c}{\rm{N}}\\ {\rm{n}}\end{array})}$$

where, N is the total number of miRNAs in the bovine reference genome, n is the number of miRNAs interacting with mRNAs (protein-coding), K is the total number of miRNAs interacting with lncRNAs, and c is the number of miRNAs shared between a ceRNA pair. All *P*-values were subjected to the false discovery rate (FDR) correction^98^.

## Electronic supplementary material

Supplementary Information 

Supplementary Table S3

Supplementary Table S4

Supplementary Table S5

Supplementary Table S6

Supplementary Table S8
