# Supplementary material for: Using RNA sequencing to identify putative competing endogenous RNAs (ceRNAs) potentially regulating fat metabolism in bovine liver
Source: Sci Rep. 2017 Jul 25;7:6396. doi: 10.1038/s41598-017-06634-w (PMC5527063; doi:10.1038/s41598-017-06634-w)
Supplement: Supplementary file 1 — Supplementary Information [file 41598_2017_6634_MOESM1_ESM.pdf]

# Using RNA sequencing to identify putative competing endogenous RNAs (ceRNAs) potentially regulating fat metabolism in bovine liver

Ruobing Liang[1,3], Bo Han[1,3], Qian Li[2], Yuwei Yuan[1], Jianguo Li[2], Dongxiao Sun[1*]

[1] Department of Animal Genetics, Breeding and Reproduction, College of Animal Science and Technology, Key Laboratory of Animal Genetics and Breeding of Ministry of Agriculture, National Engineering Laboratory of Animal Breeding, China Agricultural University, Beijing 100193, China.

[2] Department of Animal Production and Environmental Control, College of Animal Science and Technology, Hebei Agricultural University, Baoding 071001, China.

[*]Corresponding author

Email: sundx@cau.edu.cn

[3]These authors contributed equally to this work.

**Supplementary Table S1: The basic statistics for lncRNA& mRNA sequencing data.**

| Sample name | A_1 | A_2 | A_3 | B_1 | B_2 | B_3 | C_1 | C_2 | C_3 |
|---|---|---|---|---|---|---|---|---|---|
| Raw reads | 86193692 | 99986546 | 88265716 | 89354306 | 94662560 | 89655958 | 86962770 | 85912968 | 80470996 |
| Clean reads | 83667860 | 97780170 | 85888072 | 87275100 | 92522210 | 86817366 | 84045138 | 83760436 | 78622660 |
| clean bases(Gb) | 10.46 | 12.22 | 10.74 | 10.91 | 11.57 | 10.85 | 10.51 | 10.47 | 9.83 |
| Error rate(%) | 0.04 | 0.04 | 0.03 | 0.03 | 0.04 | 0.03 | 0.04 | 0.04 | 0.04 |
| Q20(%) | 92.5 | 93.24 | 93.47 | 93.55 | 93.43 | 93.56 | 92.59 | 93.03 | 92.8 |
| Q30(%) | 86.55 | 87.69 | 88.06 | 88.19 | 88.01 | 88.2 | 86.46 | 87.26 | 86.89 |
| GC content(%) | 47.97 | 49.57 | 48.72 | 48.26 | 48.98 | 48.59 | 50.39 | 48.26 | 48.68 |

Q20: the proportion of bases with a phred base quality score greater than 20; Q30: the proportion of bases with a phred base quality score greater than 30; A, B, or C represents the different individual; 1, 2, or 3 represents dry period, early lactation, or peak of lactation.

**Continued Table S1: The basic statistics for small RNA sequencing data.**

| Reads Type | A_1 | A_2 | A_3 | B_1 | B_2 | B_3 | C_1 | C_2 | C_3 |
|---|---|---|---|---|---|---|---|---|---|
| Total reads number | 10639875 | 11311351 | 12213873 | 15107016 | 13896378 | 10277358 | 13916795 | 12140340 | 12948524 |
| Low quality | 5737 | 6166 | 6581 | 8016 | 7644 | 9457 | 7498 | 6413 | 6856 |
| Adaptor3 null | 76604 | 84929 | 93449 | 118748 | 109346 | 79197 | 102389 | 92915 | 100471 |
| Insert null | 366417 | 494875 | 663170 | 694980 | 803895 | 306618 | 470220 | 509835 | 682186 |
| 5' adaptor contaminants | 6908 | 6643 | 7897 | 7368 | 8417 | 4881 | 6225 | 7088 | 8314 |
| size < 18 nt | 88201 | 79657 | 100494 | 114330 | 121052 | 151253 | 73028 | 97281 | 91171 |
| size > 31 nt | 13844 | 14793 | 22805 | 29595 | 16337 | 27368 | 15774 | 16738 | 23748 |
| polyA | 547 | 664 | 537 | 462 | 728 | 261 | 442 | 591 | 420 |
| HighQuality (18 nt <=size <= 31 nt) | 10081617 | 10623624 | 11318940 | 14133517 | 12828959 | 9698323 | 13241219 | 11409479 | 12035358 |
| Clean base(Gb) | 0.50408085 | 0.5311812 | 0.565947 | 0.70667585 | 0.64144795 | 0.48491615 | 0.66206095 | 0.57047395 | 0.6017679 |

A, B, or C represents the different individual; 1, 2, or 3 represents dry period, early lactation, or peak of lactation.

**Supplementary Table S2: Correlation coefficient ($r^2$) of the mRNA& lncRNA sequencing data among the three individual cows in each period**

| Sample | A_1 | B_1 | C_1 | A_2 | B_2 | C_2 | A_3 | B_3 | C_3 |
|---|---|---|---|---|---|---|---|---|---|
| A_1 | 1 | 0.96 | 0.95 | 0.95 | 0.94 | 0.94 | 0.96 | 0.94 | 0.94 |
| B_1 | | 1 | 0.97 | 0.96 | 0.96 | 0.96 | 0.97 | 0.96 | 0.96 |
| C_1 | | | 1 | 0.96 | 0.96 | 0.97 | 0.97 | 0.95 | 0.98 |
| A_2 | | | | 1 | 0.96 | 0.97 | 0.98 | 0.95 | 0.97 |
| B_2 | | | | | 1 | 0.97 | 0.96 | 0.96 | 0.96 |
| C_2 | | | | | | 1 | 0.96 | 0.96 | 0.98 |
| A_3 | | | | | | | 1 | 0.95 | 0.97 |
| B_3 | | | | | | | | 1 | 0.95 |
| C_3 | | | | | | | | | 1 |

A, B, or C represents the different individual; 1, 2, or 3 represents dry period, early lactation, or peak of lactation.

**Continued Table S2: Correlation coefficient ($r^2$) of the miRNA sequencing data among the three individual cows in each period**

| Sample | A_1 | B_1 | C_1 | A_2 | B_2 | C_2 | A_3 | B_3 | C_3 |
|---|---|---|---|---|---|---|---|---|---|
| A_1 | 1 | 0.99 | 0.99 | 1.00 | 0.98 | 0.98 | 1.00 | 0.98 | 0.99 |
| B_1 | | 1 | 0.99 | 0.99 | 0.99 | 0.99 | 0.99 | 0.99 | 1.00 |
| C_1 | | | 1 | 0.99 | 0.99 | 0.99 | 0.98 | 0.99 | 0.99 |
| A_2 | | | | 1 | 0.98 | 0.98 | 1.00 | 0.98 | 0.99 |
| B_2 | | | | | 1 | 1.00 | 0.98 | 0.99 | 0.99 |
| C_2 | | | | | | 1 | 0.97 | 0.99 | 0.99 |
| A_3 | | | | | | | 1 | 0.97 | 0.99 |
| B_3 | | | | | | | | 1 | 0.98 |
| C_3 | | | | | | | | | 1 |

A, B, or C represents the different individual; 1, 2, or 3 represents dry period, early lactation, or peak of lactation.

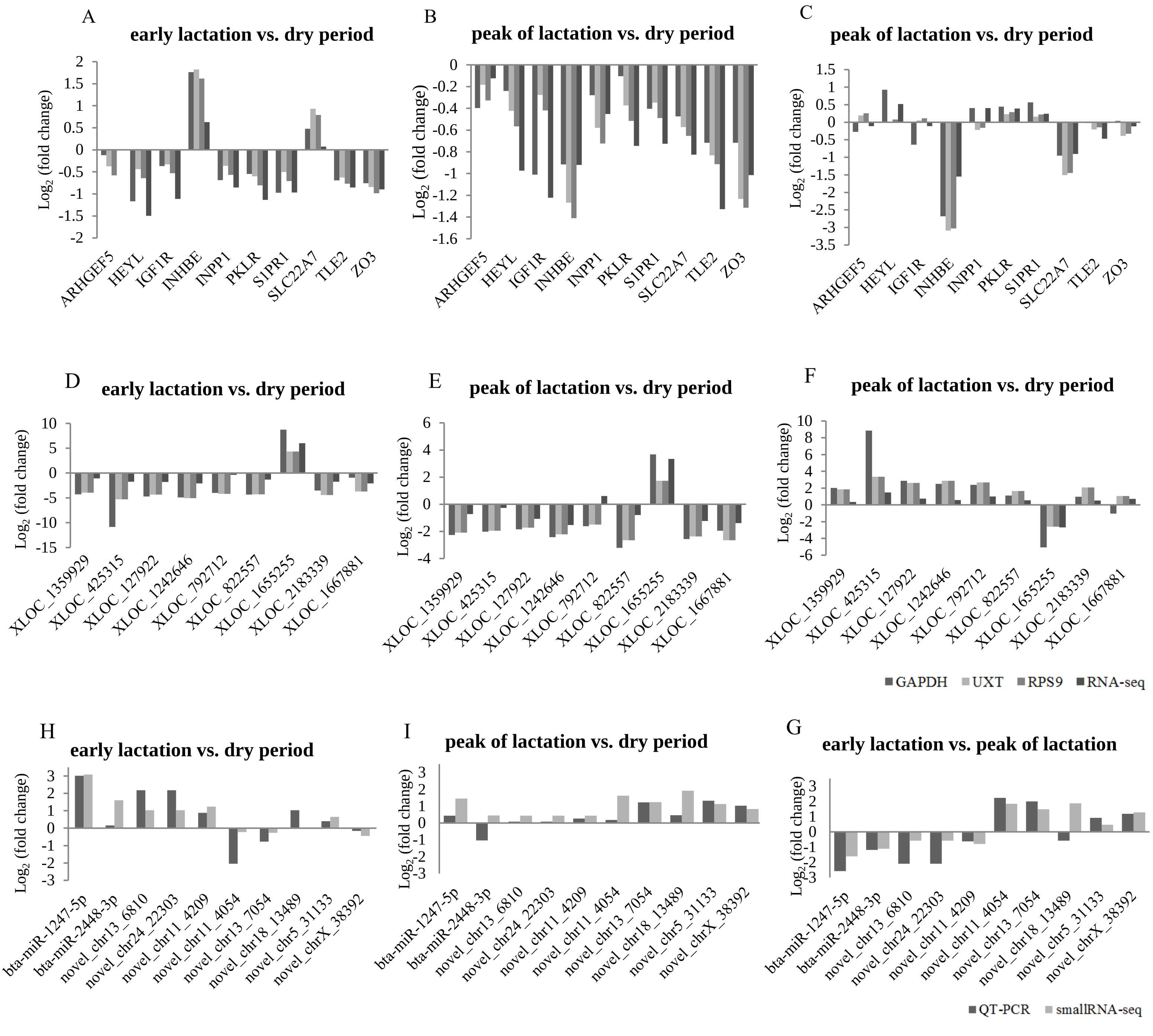


**Supplementary Figure S1. Comparison of the expression change levels of 10 mRNAs, nine lncRNAs, and 10 miRNAs randomly selected in the three periods between sequencing and qRT-PCR.** A, B, C shows 10 mRNAs expression fold changes in every two periods comparison, y-axis shows the $\log_2$ (ratio of mRNA levels between two periods) measured by qRT-PCR (normalized by *GAPDH*, *UXT* and *RPS9*) and RNA-seq, respectively; D, E, F shows nine lncRNAs expression fold changes in every two periods comparison, y-axis shows the $\log_2$ (ratio of lncRNA levels between two periods) measured by qRT-PCR (normalized by *GAPDH*, *UXT* and *RPS9*) and RNA-seq, respectively; and G, H, I shows 10 miRNAs expression fold changes in every two periods comparison, y-axis shows the $\log_2$ (ratio of miRNA levels between two periods) measured by qRT-PCR (normalized by snoRNA U6) and small RNA-seq, respectively.

**Supplementary Table S7: ceRNA pairs identified during dry period, early lactation, and peak of lactation.**

| Comparison | lncRNA_ID | shared_miRNA | MiRNA up/down | mRNA_ID | gene_name | p_value | FDR |
|---|---|---|---|---|---|---|---|
| Early lactation vs. dry period | XLOC_822557 | novel_chr21_19609,bta-miR-1247-5p | miRNA_up | ENSBTAT00000002373 | ARHGEF5 | 8.43E-07 | 1.12E-05 |
| | XLOC_1359929 | novel_chr13_6810,novel_chr24_22303 | miRNA_up | ENSBTAT00000028690 | IGF1R | 8.43E-07 | 1.05E-05 |
| | XLOC_1667881 | novel_chr24_22303,novel_chr13_6810 | miRNA_up | ENSBTAT00000034627 | CX3CL1 | 8.43E-07 | 1.01E-05 |
| | XLOC_1359929 | novel_chr24_22303,novel_chr13_6810 | miRNA_up | ENSBTAT00000034627 | CX3CL1 | 8.43E-07 | 8.17E-06 |
| | XLOC_425315 | novel_chr6_33592,bta-miR-2448-3p | miRNA_up | ENSBTAT00000009977 | INPP1 | 8.43E-07 | 8.02E-06 |
| | XLOC_1667881 | novel_chr13_6810,novel_chr24_22303 | miRNA_up | ENSBTAT00000028690 | IGF1R | 8.43E-07 | 7.50E-06 |
| | XLOC_1667881 | novel_chr13_6810,novel_chr24_22303 | miRNA_up | ENSBTAT00000018241 | GPR37 | 8.43E-07 | 6.64E-06 |
| | XLOC_822557 | bta-miR-1247-5p,novel_chr21_19609 | miRNA_up | ENSBTAT00000003229 | TNS1 | 8.43E-07 | 6.63E-06 |
| | XLOC_1359929 | novel_chr13_6810,novel_chr24_22303 | miRNA_up | ENSBTAT00000018241 | GPR37 | 8.43E-07 | 6.41E-06 |
| | XLOC_822557 | bta-miR-1247-5p,novel_chr21_19609 | miRNA_up | ENSBTAT00000015668 | COL6A1 | 2.53E-06 | 1.52E-05 |
| | XLOC_822557 | bta-miR-1247-5p,novel_chr21_19609 | miRNA_up | ENSBTAT00000061325 | AHDC1 | 8.43E-06 | 3.49E-05 |
| | XLOC_127922 | novel_chr6_33592,bta-miR-2448-3p | miRNA_up | ENSBTAT00000009977 | INPP1 | 8.43E-06 | 3.48E-05 |
| | XLOC_1242646 | novel_chr11_4209 | miRNA_up | ENSBTAT00000020610 | FADS2 | 0.000648929 | 0.002117922 |
| | XLOC_1242646 | novel_chr11_4209 | miRNA_up | ENSBTAT00000025788 | CGN1 | 0.000648929 | 0.001241418 |
| | XLOC_1242646 | novel_chr11_4209 | miRNA_up | ENSBTAT00000018649 | CGN1 | 0.000648929 | 0.001238117 |
| | XLOC_1242646 | novel_chr11_4209 | miRNA_up | ENSBTAT00000007861 | S1PR1 | 0.001297859 | 0.001979818 |
| Peak of lactation vs. dry period | XLOC_2183339 | novel_chr11_4054,novel_chr13_7054 | miRNA_up | ENSBTAT00000002076 | WIPF1 | 8.43E-07 | 1.20E-05 |
| | XLOC_2183339 | novel_chr11_4054,novel_chr13_7054 | miRNA_up | ENSBTAT00000001534 | TLE2 | 2.53E-06 | 3.00E-05 |
| | XLOC_2183339 | novel_chr13_7054,novel_chr11_4054 | miRNA_up | ENSBTAT00000009497 | CASKIN2 | 1.77E-05 | 0.000123531 |
| | XLOC_2183339 | novel_chr13_7054,novel_chr11_4054 | miRNA_up | ENSBTAT00000049235 | DDAH1 | 1.77E-05 | 0.000122754 |
| | XLOC_2183339 | novel_chr11_4054,novel_chr13_7054 | miRNA_up | ENSBTAT00000019489 | ZFHX3 | 1.77E-05 | 0.000121718 |
| | XLOC_1242646 | novel_chr13_7054 | miRNA_up | ENSBTAT00000011453 | HEYL | 0.001297859 | 0.004330871 |
| | XLOC_1242646 | novel_chr13_7054 | miRNA_up | ENSBTAT00000018649 | CGN1 | 0.001297859 | 0.004030836 |
| | XLOC_1242646 | novel_chr13_7054 | miRNA_up | ENSBTAT00000025788 | CGN1 | 0.001297859 | 0.003962106 |

| | | | | | | |
|---|---|---|---|---|---|---|
| XLOC_1242646 | novel_chr13_7054 | miRNA_up | ENSBTAT00000034627 | CX3CL1 | 0.001297859 | 0.003731857 |
| XLOC_2183339 | novel_chr13_7054 | miRNA_up | ENSBTAT00000018649 | CGN1 | 0.001297859 | 0.003693623 |
| XLOC_2183339 | novel_chr13_7054 | miRNA_up | ENSBTAT00000025788 | CGN1 | 0.001297859 | 0.003691103 |
| XLOC_2183339 | novel_chr13_7054 | miRNA_up | ENSBTAT00000011453 | HEYL | 0.001297859 | 0.003500123 |
| XLOC_2183339 | novel_chr11_4054 | miRNA_up | ENSBTAT00000029841 | SFRP2 | 0.001297859 | 0.003398686 |
| XLOC_2183339 | novel_chr13_7054 | miRNA_up | ENSBTAT00000034627 | CX3CL1 | 0.001297859 | 0.003397779 |
| XLOC_054981 | novel_chr18_13489 | miRNA_up | ENSBTAT00000046947 | PKLR | 0.001946788 | 0.004788902 |
| XLOC_054981 | novel_chr18_13489 | miRNA_up | ENSBTAT00000028521 | HEG1 | 0.001946788 | 0.004774452 |
| XLOC_054981 | bta-miR-2887 | miRNA_up | ENSBTAT00000008663 | INHBE | 0.001946788 | 0.004709402 |
| XLOC_054981 | novel_chr18_13489 | miRNA_up | ENSBTAT00000018241 | GPR37 | 0.001946788 | 0.004651971 |
| XLOC_2183339 | novel_chr11_4054 | miRNA_up | ENSBTAT00000063189 | TPC3 | 0.002594874 | 0.005769479 |
| XLOC_2183339 | novel_chr13_7054 | miRNA_up | ENSBTAT00000011517 | SAMD11 | 0.002594874 | 0.005757275 |
| XLOC_1242646 | novel_chr13_7054 | miRNA_up | ENSBTAT00000011517 | SAMD11 | 0.002594874 | 0.00572928 |
| XLOC_2183339 | novel_chr11_4054 | miRNA_up | ENSBTAT00000065036 | BCOR | 0.002594874 | 0.005700368 |
| XLOC_1242646 | novel_chr5_31133 | miRNA_up | ENSBTAT00000008336 | SLC22A7 | 0.002594874 | 0.005580781 |
| XLOC_2183339 | novel_chr11_4054 | miRNA_up | ENSBTAT00000011846 | - | 0.002594874 | 0.005538246 |
| XLOC_1242646 | novel_chr13_7054 | miRNA_up | ENSBTAT00000002076 | WIPF1 | 0.002594874 | 0.005476775 |
| XLOC_2183339 | novel_chr13_7054 | miRNA_up | ENSBTAT00000035742 | ZFP36L1 | 0.002594874 | 0.005448703 |
| XLOC_1242646 | novel_chr13_7054 | miRNA_up | ENSBTAT00000035742 | ZFP36L1 | 0.002594874 | 0.005380762 |
| XLOC_2183339 | novel_chr11_4054 | miRNA_up | ENSBTAT00000023515 | NOS3 | 0.003891047 | 0.00686203 |
| XLOC_054981 | novel_chr18_13489 | miRNA_up | ENSBTAT00000021480 | ZNF608 | 0.003891047 | 0.006847501 |
| XLOC_054981 | novel_chr18_13489 | miRNA_up | ENSBTAT00000027940 | NGFR | 0.003891047 | 0.006837597 |
| XLOC_054981 | novel_chr5_31133 | miRNA_up | ENSBTAT00000008336 | SLC22A7 | 0.003891047 | 0.006701767 |
| XLOC_2183339 | novel_chr13_7054 | miRNA_up | ENSBTAT00000033431 | NFIA | 0.003891047 | 0.00650726 |
| XLOC_054981 | bta-miR-2887 | miRNA_up | ENSBTAT00000013942 | FGFR4 | 0.003891047 | 0.006473791 |
| XLOC_1242646 | novel_chr13_7054 | miRNA_up | ENSBTAT00000033431 | NFIA | 0.003891047 | 0.006380171 |
| XLOC_1242646 | novel_chr13_7054 | miRNA_up | ENSBTAT00000001534 | TLE2 | 0.003891047 | 0.006374018 |
| XLOC_2183339 | novel_chr11_4054 | miRNA_up | ENSBTAT00000015828 | PLEC | 0.005186378 | 0.00775438 |

| | | | | | | | |
|---|---|---|---|---|---|---|---|
| | XLOC_1242646 | novel_chr5_31133 | miRNA_up | ENSBTAT00000028690 | IGF1R | 0.005186378 | 0.007546511 |
| | XLOC_1242646 | novel_chr5_31133 | miRNA_up | ENSBTAT00000022744 | HSPG2 | 0.005186378 | 0.007488562 |
| | XLOC_054981 | novel_chr18_13489 | miRNA_up | ENSBTAT00000033431 | NFIA | 0.00583278 | 0.008023816 |
| | XLOC_054981 | novel_chr18_13489 | miRNA_up | ENSBTAT00000002427 | PPP2R2B | 0.00583278 | 0.007929014 |
| | XLOC_054981 | novel_chr18_13489 | miRNA_up | ENSBTAT00000000176 | LRFN3 | 0.00583278 | 0.007928703 |
| | XLOC_2183339 | novel_chr11_4054 | miRNA_up | ENSBTAT00000024404 | ZO3 | 0.006480865 | 0.008542226 |
| | XLOC_2183339 | novel_chr11_4054 | miRNA_up | ENSBTAT00000004201 | IQSEC1 | 0.006480865 | 0.008487475 |
| | XLOC_054981 | novel_chr18_13489 | miRNA_up | ENSBTAT00000017323 | ARNTL | 0.007771988 | 0.009718044 |
| | XLOC_054981 | novel_chr5_31133 | miRNA_up | ENSBTAT00000022744 | HSPG2 | 0.007771988 | 0.009658766 |
| | XLOC_054981 | novel_chr5_31133 | miRNA_up | ENSBTAT00000028690 | IGF1R | 0.007771988 | 0.009481362 |
| | XLOC_2183339 | novel_chr11_4054 | miRNA_up | ENSBTAT00000003229 | TNS1 | 0.00777451 | 0.00937461 |
| | XLOC_1242646 | novel_chr13_7054 | miRNA_up | ENSBTAT00000049235 | DDAH1 | 0.009067312 | 0.010754686 |
| | XLOC_1242646 | novel_chr13_7054 | miRNA_up | ENSBTAT00000009497 | CASKIN2 | 0.009067312 | 0.010687278 |
| | XLOC_1242646 | novel_chr13_7054 | miRNA_up | ENSBTAT00000019489 | ZFHX3 | 0.009067312 | 0.010678777 |
| | XLOC_1242646 | novel_chr13_7054 | miRNA_up | ENSBTAT00000031875 | PRR12 | 0.01035927 | 0.011669388 |
| | XLOC_2183339 | novel_chr11_4054 | miRNA_up | ENSBTAT00000013284 | EGR1 | 0.01035927 | 0.011656396 |
| | XLOC_2183339 | novel_chr11_4054 | miRNA_up | ENSBTAT00000024256 | SIPA1L3 | 0.01035927 | 0.011589121 |
| | XLOC_2183339 | novel_chr13_7054 | miRNA_up | ENSBTAT00000031875 | PRR12 | 0.01035927 | 0.011575913 |
| | XLOC_2183339 | novel_chr11_4054 | miRNA_up | ENSBTAT00000007134 | GATA4 | 0.01035927 | 0.011575229 |
| | XLOC_054981 | bta-miR-2887 | miRNA_up | ENSBTAT00000003229 | TNS1 | 0.01164284 | 0.012824171 |
| | XLOC_2183339 | novel_chr11_4054 | miRNA_up | ENSBTAT00000002375 | PLXND1 | 0.01165039 | 0.0127609 |
| | XLOC_054981 | novel_chr18_13489 | miRNA_up | ENSBTAT00000019489 | ZFHX3 | 0.01357448 | 0.014562026 |
| | XLOC_054981 | novel_chr18_13489 | miRNA_up | ENSBTAT00000028865 | TNRC18 | 0.0193543 | 0.019862799 |
| Peak of lactation vs. early lactation | XLOC_792712 | novel_chr21_19609,bta-miR-1247-5p | miRNA_down | ENSBTAT00000013221 | FAM107B | 8.43E-06 | 4.40E-05 |
| | XLOC_1655255 | novel_chrX_38392 | miRNA_up | ENSBTAT00000010711 | PKIA | 0.000648929 | 0.001652694 |
| | XLOC_1655255 | novel_chrX_38392 | miRNA_up | ENSBTAT00000016009 | CCDC167 | 0.000648929 | 0.001636175 |
| | XLOC_1655255 | novel_chrX_38392 | miRNA_up | ENSBTAT00000019772 | NR0B2 | 0.002595717 | 0.005046777 |
| | XLOC_1655255 | novel_chrX_38392 | miRNA_up | ENSBTAT00000022843 | C20ORF82 | 0.003893576 | 0.006644547 |

**Supplementary Table S9: Expression of lipid metabolic genes during dry period, early lactation, and peak of lactation.**

| Metabolic processes | Gene name | dry period (FPKM) | early lactation (FPKM) | peak of lactation (FPKM) |
|---|---|---|---|---|
| | *SREBF1* | 10.61 | 7.39 | 6.55 |
| | *SREBF2* | 7.53 | 5.13 | 4.98 |
| | *PPARA* | 40.75 | 32.49 | 34.45 |
| lipid transport | *APOA1* | 185.79 | 1161.93 | 304.93 |
| | *APOA2* | 10523.09 | 7078.26 | 9339.80 |
| | *APOC3* | 1880.12 | 1426.49 | 1648.34 |
| | *APOA5* | 273.51 | 471.99 | 446.11 |
| | *PLTP* | 4.83 | 7.97 | 5.04 |
| lipogenesis | *ME* | 6.14 | 7.75 | 5.87 |
| | *SCD* | 41.08 | 22.89 | 79.77 |
| | *FADS2* | 383.45 | 138.22 | 325.31 |
| | *FASN* | 2.81 | 1.39 | 1.91 |
| | *ACACA* | 4.84 | 4.44 | 6.58 |
| cholesterol metabolism | *CYP7A1* | 123.74 | 364.46 | 327.11 |
| | *CYP8B1* | 156.41 | 122.03 | 137.05 |
| | *CYP27A* | 184.58 | 230.98 | 294.66 |
| | *NRIH3* | 48.83 | 54.86 | 53.11 |
| fatty acid transport | *DBI* | 488.93 | 771.02 | 717.47 |
| | *FABP1* | 987.53 | 1303.89 | 1089.08 |
| | *FABP3* | 1.66 | 2.95 | 1.09 |
| | *CD36* | 14.85 | 30.70 | 18.61 |
| | *SLC27A2* | 180.02 | 249.44 | 227.39 |
| | *LPL* | 5.10 | 3.66 | 3.97 |
| | *ACSL1* | 80.75 | 183.16 | 110.58 |
| | *OLR1* | 0.87 | 1.31 | 1.20 |
| fatty acid oxidation | *EHHADH* | 229.85 | 291.60 | 274.13 |
| | *ACAA1* | 429.31 | 547.26 | 557.52 |
| | *SCP2* | 720.69 | 878.03 | 1070.84 |
| | *ACOX* | 157.09 | 215.90 | 220.89 |
| | *CPT1A* | 57.72 | 93.99 | 91.95 |
| | *CPT1B* | 0.80 | 6.92 | 2.36 |
| | *CPT1C* | 0.25 | 0.31 | 0.32 |
| | *CPT2* | 54.03 | 79.21 | 69.61 |
| | *ACADL* | 74.43 | 88.24 | 82.54 |
| | *ACADM* | 558.43 | 850.58 | 786.05 |

The differentially expressed genes were marked in red.

**Supplementary Table S10: Primers of the differentially expressed mRNAs, lncRNAs, and miRNAs randomly selected for qRT-PCR.**

| RNA | Name | Forward primer sequence(5'-3') | Reverse primer sequence(5'-3') | Amplicon (bp) | Tm (°C) |
|---|---|---|---|---|---|
| mRNA | *ARHGEF5* | GTCCGTGGGGAAAAGTGTGA | GGCATAGCCAAGGCTGAGAT | 158 | 60 |
| | *HEYL* | AGCCCTTCACAAATGCAAGC | TTCTCAAAGGCAGTGGGGAC | 119 | 60 |
| | *IGF1R* | CGCTTGTCCCAAAACCGAAG | CGATCTGCATGACCTCCCTC | 137 | 60 |
| | *INHBE* | GGACTACAGCTGACCAGTCG | GGACAGGTGGAAGGTGAGTG | 186 | 60 |
| | *INPP1* | TTGGGAATCTGGGTGGATCC | GGCACCCCTGTCTGTATGTC | 146 | 60 |
| | *PKLR* | CATCGACTCTGAGCCTGTGG | CTCCCGGATGTTAGCGATGG | 178 | 60 |
| | *S1PR1* | AGCCCTGTCTCGGACTATGT | CGCGCTGATCTTCAGCTTTC | 78 | 60 |
| | *SLC22A7* | CTGTTGGCACTGATCGGCTA | GATTCAGGCACCCACCAGAG | 107 | 60 |
| | *TLE2* | GAGTGTGGTGGAAGAGGAGC | CGCTCTTGTCCTCGTCACTT | 107 | 60 |
| | *ZO3* | CTGGTGAATATCCCTCCGGC | CTGGTGTCTGGACTCTGCTG | 143 | 60 |
| lncRNA | *XLOC_1359929* | CAGGCCACATGCTCGGTAA | ACTCCTGCCTCTGTCACATC | 204 | 60 |
| | *XLOC_425315* | TGCATTCGAGAATGGCACCT | TAGCGTCATGGAGGGGTGTA | 106 | 60 |
| | *XLOC_127922* | CCCATTGGACAACTCTGGCT | ACCAGAAGTTTGAAGAGTGTCCTG | 177 | 60 |
| | *XLOC_1242646* | TTCATCTTCAGCCTGGGTCG | GTTTGGGACTGCAGGTCTGT | 135 | 60 |
| | *XLOC_792712* | AAGTTCTCACCTTGGCACCTC | TAATAACAACCAGCCTCCCTCG | 168 | 60 |
| | *XLOC_822557* | AGGGCCTGATGGGCTTACTA | AACAGTCCTTCCACTGGTGC | 152 | 60 |
| | *XLOC_1655255* | TAAGACCCAGCGTCGAATGG | AGGCTCAGTTTCCCTGCATC | 123 | 60 |
| | *XLOC_2183339* | CCTGGCTGGGTTCCTCTTTT | CAGAGAAGGACCCCAAGCAG | 195 | 60 |
| | *XLOC_1667881* | AGAGTCAGGAGATGGGGCTT | GTGCTCAGCTGTGTCTGACT | 126 | 60 |
| Housekeeping | *GAPDH* | AGATGGTGAAGGTCGGAGTG | CCTTTCCATTGATGACGAGC | 205 | 60 |
| | *UXT* | TGTGGCCCTTGGATATGGTT | GGTTGTCGCTGAGCTCTGTG | 101 | 60 |
| | *RPS9* | CCTCGACCAAGAGCTGAAG | CCTCGACCAAGAGCTGAAG | 54 | 60 |
| miRNA | bta-miR-1247-5p | TGAACGTCGGGACTGGAGC | The reverse primers for miRNA validation were supplied in the box of the miRcute Plus miRNA qPCR Detection Kit (TIANGEN, Beijing, | | 60 |
| | bta-miR-2448-3p | GCGGATACAACCAAACACGG | | | 60 |
| | novel_chr13_6810 | GGTCCCCTTCAACCAGCTGT | | | 60 |
| | novel_chr24_22303 | GGTCCCCTTCAACCAGCTGT | | | 60 |

| | | | | |
|---|---|---|---|---|
| | novel_chr11_4209 | GGTTCGATCCCACTTCTGACAC | China). | 60 |
| | novel_chr11_4054 | ATAATAATAGCGGGGCCGGG | | 60 |
| | novel_chr13_7054 | TATATAGTGGGGGGGCGGC | | 60 |
| | novel_chr18_13489 | ATATATACGCGCGCCGGTG | | 60 |
| | novel_chr5_31133 | TATATACCCTGGGAGCCCGG | | 60 |
| | novel_chrX_38392 | TATTATATACGGCGGCGGCG | | 60 |
| | U6 snoRNA (reference) | has-U6 (CD201-0145,TIANGEN) | | 60 |
